# Home Visits for Children With Asthma Reduce Medicaid Costs

**DOI:** 10.5888/pcd17.190288

**Published:** 2020-02-06

**Authors:** Erica T. Marshall, Jing Guo, Elizabeth Flood, Megan T. Sandel, Matthew D. Sadof, Jean M. Zotter

**Affiliations:** 1 Massachusetts Department of Public Health, Boston, Massachusetts; 2 JSI Research and Training Institute, Boston, Massachusetts; 3 Boston University School of Medicine, Boston, Massachusetts; 4 University of Massachusetts Medical School-Baystate, Springfield, Massachusetts

## Abstract

We conducted a multicomponent, low-cost, home intervention for children with uncontrolled asthma, the Reducing Ethnic/Racial Asthma Disparities in Youth (READY) study, to evaluate its effect on health outcomes and its return on investment. From 2009 through 2014 the study enrolled 289 children aged 2 to 13 years with uncontrolled asthma and their adult caregivers in Boston and Springfield, Massachusetts. Community health workers (CHWs) led in-home asthma management and environmental trigger remediation education over 5 visits spanning 6 months. Asthma health outcomes and indoor environment data were collected via survey, and health use costs were accessed through Massachusetts Medicaid (MassHealth). Results showed significant improvements in asthma control, health care use, and environmental trigger reduction and a positive return on investment (1.34) for participants who had 2 or more emergency department visits 1 year prior to the first home visit. The CHW asthma home visiting intervention improved trigger management, clinical outcomes, and Medicaid cost savings, demonstrating that asthma home visits improve health quality and reduce costs.

## Introduction

The effectiveness of home visits by community health workers (CHWs) to children with asthma in improving health outcomes for high-risk populations is established in the literature (1). However, that evidence-based model of care has not been widely adopted by health care systems, mainly because of limited coverage by health care payers (2). Although many studies have estimated a positive return on investment (ROI) for these home visits, few have used verified cost and use data from participants’ medical claims. This absence of data is a barrier to widespread payer reimbursement (3–5). Our study, Reducing Ethnic/Racial Asthma Disparities in Youth (READY), used insurance claims data to determine the potential cost savings for Massachusetts Medicaid (MassHealth) of integrating a CHW-led asthma home visiting program into the medical home model of care. The READY study was approved by the Massachusetts Department of Public Health (MDPH) Institutional Review Board (Approval # 00000701).

## Purpose and Objectives

The READY study evaluated the effect of a CHW-led home intervention on improving outcomes for children with uncontrolled asthma and the direct and indirect costs associated with the intervention, with a particular focus on Medicaid-enrolled children.

## Evaluation Methods

### Study population, structure, and sample

The READY study was administered by the Massachusetts Department of Public Health (MDPH) with its clinical partners, Boston Medical Center and Baystate Medical Center, as study sites, from September 2009 to April 2014. Both partners serve predominantly low-income Hispanic and non-Hispanic black patient populations. To be eligible for the study, a child had to meet at least 1 of 4 criteria: 1) be aged 2 to 13 years and currently a patient of either study site; 2) have uncontrolled, persistent asthma (6); 3) have had 1 emergency department visit, hospitalization, or unscheduled office visit for asthma in the past year; or 4) was on a course of oral steroids in the past year. Recruitment took place among families with children who were asthma patients at the 2 clinical sites, Boston Medical Center and Baystate Medical Center, or their affiliated health centers. READY enrolled 289 pairs of children and their adult caregivers. Two hundred and fifty-four child–caregiver pairs (87.9%) received an initial visit from a CHW, 170 pairs (58.8%) completed all 5 home visits, and 136 pairs (47.1%) completed a follow-up telephone call 1 year post-enrollment.

READY modified the evidence-based protocols from the Seattle–King County Healthy Homes Project (7) for implementation to integrate CHWs into the medical home team. The READY protocol consisted of 5 home visits by a CHW over 6 months, plus a follow-up telephone call at 12 months. CHWs provided asthma self-management education, environmental trigger remediation education, and low-cost trigger remediation supplies (eg, HEPA vacuum cleaners, dust mite covers) (Table 1, Table 2).

**Table 1:** Intervention Timeline, Reducing Ethnic/Racial Asthma Disparities in Youth (READY) Study, 2009–2014

Intervention activity	Visit Number^a^				
	1	2	3	4	5

Month of visit^b^	0	0.5	1	2	6
**Activity**					
**General**					
Project overview	X	X	—	—	—
Set asthma and home priorities and make plan	—	X	—	—	—
Revisit family priorities	—	—	X	X	X
**Environmental assessment/education**					
Home environmental walk-through	—	X	—	—	—
Pest education, develop plan	—	—	X	—	—
Dust mite education, develop plan	—	X	—	—	—
Pets education, develop plan	—	X	—	—	—
Mold education, develop plan	—	X	—	—	—
Dust control education, develop plan	—	—	—	X	—
Tobacco education, develop plan	—	—	X	(X)	—
Reassessment of environmental plans	—	—	X	X	(X)
**Social assessment/education**					
Urgent needs assessment, link with services	X	X	—	—	—
Revisit needs	—	X	(X)	(X)	(X)
**Asthma assessment/education**					
Asthma basics	X	(X)	—	—	—
Asthma management education	—	—	X	X	X
Asthma trigger identification and avoidance	—	X	X	X	—
Asthma care understanding and assessments of barriers to optimal asthma control	X	X	X	X	X

Abbreviation: —, no activity at a visit; X, activity occurred at a visit; (X), activity may have occurred at a visit (at community health worker’s discretion).

a Five visits were made over a 6-month period.

b Refers to the month of the 6-month study. Some visits were conducted mid month (ie, month 0.5).

**Table 2:** Evaluation Timeline, Reducing Ethnic/Racial Asthma Disparities in Youth (READY) Study, 2009–2014

Data Collection and Evaluation	Visit Number^a^				
	1	2	3	4	5

Month of Visit^b^	0	0.5	1	2	4–6
Consent form (READY and MassHealth permission to share information)	X	—	—	—	—
Home environmental checklist (from Seattle Home Study [7])	—	X	—	—	X
Encounter form (to document CHW actions in home)	X	X	X	X	X
Environmental action plan	—	X	X	X	X
Asthma 2-week recall symptom checklist (from Seattle Home Study [7])	X	X	X	X	X
Baseline survey 1 (asthma data including the Pediatric Asthma Caregiver Quality of Life [14])	X	—	—	—	X
Baseline survey 2 (parental expectations, asthma triggers)	—	X	—	—	X

Abbreviation: —, no activity at a visit; X, activity occurred at a visit; (X), activity may have occurred at a visit (at community health worker’s discretion).

a Five visits were made over a 6-month period.

b Refers to the month of the 6-month study. Some visits were conducted mid month (ie, month 0.5).

The sample size was based on results from the Massachusetts Behavioral Risk Factor Surveillance System 2006 Child Call-Back Survey, a Massachusetts Department of Public Health database, in which respondents self-reported asthma symptoms in response to the question, “During the past 30 days, on how many days did you have any symptoms of asthma?” A sample size of 260 was deemed necessary to detect a minimum difference in the average number of self-reported asthma symptom days among participants at visit 1 compared with visit 5 (post-intervention), with 80% power and a type 1 error of 5%.

### Data collection and analysis

Two data sources were used for final analyses. Asthma symptoms and health care use (ie, whether a participant had a hospitalization, emergency department visit, or an unscheduled office visit because of asthma) were collected by caregiver self-report, and environmental trigger information was collected on the basis of caregiver self-report and CHW observation at visits 1 and 5. Asthma control focused on 2 domains: 1) reducing impairment — the frequency and intensity of symptoms and functional limitations currently or recently experienced by a patient; and 2) reducing risk — the likelihood of future asthma attacks, progressive decline in lung function (or, for children, reduced lung growth), or medication side effects (8). The participants’ levels of asthma control were classified by using the National Asthma Education Prevention Program’s Expert Panel Report 3 Full Report 2007: Guidelines for the Diagnosis and Management of Asthma (8) and were based on caregiver self-reports. Data used to classify asthma control were the number of asthma symptom days, the number of nighttime awakenings, the limitation of normal activity because of asthma, and the number of times asthma rescue medications were used. MDPH developed a composite score to quantify indoor environmental asthma triggers. The score was calculated by summing 6 indicators that represented the presence of 1 or more of the 6 asthma trigger measures (dust, mold, pests, smoke, pets, and chemicals) in the home. Composite scores ranged from 0 to 6, with a lower score indicating fewer asthma triggers found in the home. Each trigger indicator score was based on the parental report of exposures to each asthma trigger in the home and the CHW’s observations of evidence of triggers. For those study participants enrolled in Medicaid, health care use costs were accessed through MassHealth claims data. All caregivers signed permission to share information (PSI) forms at enrollment, which allowed MDPH to verify MassHealth enrollment and access their children’s MassHealth claims, if enrolled. Of the 170 children who completed all 5 home visits, 128 had a signed PSI form, 112 had matched MassHealth claims during the study period, 99 had 1 year of continuous Medicaid enrollment before visit 1 (1 year prior to visit 1 was defined as the control period) and 1 year after visit 5 (1 year after visit 5 was defined as post period), and 70 children without a third-party payer were included in the analysis. Secondary cost analysis was performed among participants with higher health care use, defined as those with 2 or more emergency department claims (n = 22) in the control period. The MassHealth claims database contains claims for hospitalization, emergency department, office visit, and pharmacy. However, pharmacy claims were incomplete and were not included in the cost analysis.

We used χ^2^ tests and unpaired *t* tests to examine differences in categorical and continuous asthma outcomes between visit 1 and visit 5 from caregiver self-reports. Mann–Whitney U tests were performed to compare changes in medical expenses, and paired *t* tests were applied to evaluate differences in health care use during the control period and post period from MassHealth claims. SAS version 9.3 (SAS Institute Inc) was used to perform analyses.

ROI was based on average total preventable asthma costs per participant during the control and post periods (the total preventable asthma costs per participant were total asthma-related hospitalization costs and total asthma-related emergency department visit costs per participant) from MassHealth claims and average program costs of the READY intervention per study participant. Program costs were salary and benefits for 1 full-time equivalent CHW per clinical site, 0.2 full-time equivalent salary and benefits for a CHW supervisor per clinical site, and supplies (HEPA vacuum cleaners, mattress and pillow covers, and green cleaning products). The average participant program costs were based on an average annual caseload of 40 participants per 1 full-time equivalent CHW. ROI was calculated by using this formula:

ROI = (total preventable costs control period – total preventable costs post period)/present value of program costs

## Results

The average age of children who completed visit 1 (n = 254) was 6.2 years; 58.7% were male, 48.0% were non-Hispanic African American, 49.2% were Hispanic, and 2.8% were non-Hispanic white. Most caregivers reported their children had Medicaid as their health insurer (93.2%).

**Results from caregivers’ self-report (n = 254).** Intent-to-treat analysis using self-reports showed the average number of asthma symptom days decreased 2.97 days (from 5.26 days, standard deviation [SD] = 4.07) to 2.29 days [SD = 3.20], *P* < .001). The proportion of participants with well-controlled asthma showed an absolute increase of 22.9 percentage points (pp) (from 17.7 pp, 95% confidence interval [CI], 13.02 pp–22.41 pp to 40.6 pp, 95% CI, 33.21 pp–47.97 pp; *P* = .004). The proportion of participants who had emergency department visits (absolute decrease of 41.63 pp; from 64.57 pp [95% CI, 58.69 pp–70.45 pp] to 22.94 pp, *P* < .001), hospitalizations (absolute decrease 21.09 pp; from 26.38 pp [95% CI, 20.96 pp–31.80 pp] to 5.29 pp [95% CI, 1.93 pp–8.66 pp], *P* < .001), or used urgent health services (absolute decrease 37.37 pp; from 80.31 pp [95% CI, 75.43 pp–85.21 pp] to 42.94 pp [95% CI, 35.50 pp–50.38 pp], *P* < .001) decreased significantly between visit 1 and visit 5. The results showed significantly reduced environmental trigger exposures, such as dust, mold, pests, and chemical exposures.

**Results from participants’ MassHealth claims (n = 70).** MassHealth claims (Table 3) showed that the average number of asthma-related emergency department visits per year decreased 46% (from 0.93 to 0.50, *P* = .004) between the control period and post period. When analyses were restricted to participants with 2 or more emergency department visits during the control period, the average number of asthma related emergency department visits decreased 63%, from 2.59 visits to 0.95 visits, *P* < .001.

**Table 3:** Insurance Claims for Participants (N = 70) Covered by MassHealth (Medicaid) Related to Asthma, Reducing Racial/Ethnic Asthma Disparities in Youth (READY) Study, Massachusetts, 2009–2014

Variable	Control Period^a^	Post Period^b^	*P *Value^c^
	Mean (Standard Deviation)	Mean (Standard Deviation)	
**Participants who completed all 5 visits (n = 70)**			
**Average cost per person, $**			
Hospitalization	1,059.22 (3,199.62)	506.20 (1,948.86)	.11
Emergency department visits	534.73 (809.61)	223.41 (473.77)	.001
Office visits	473.77 (618.94)	406.17 (537.90)	.32
**Health care events, no.**			
Hospitalization	0.21 (0.66)	0.10 (0.39)	.11
Emergency department visits	0.93 (1.15)	0.50 (0.86)	.004
Office visits	3.19 (3.28)	2.96 (3.09)	.69
**Participants who completed all 5 visits and had 2 or more emergency department visits during control period (n = 22)**			
**Average cost per person, $**			
Hospitalization	2,543.76 (4,372.83)	1,243.14 (4,378.39)	.23
Emergency department	1,512.87 (828.25)	454.39 (1,206.34)	.009
Office	730.22 (868.92)	739.91 (773.25)	.52
**Health care events, no.**			
Hospitalization	0.64 (1.09)	0.32 (0.78)	.13
Emergency department visit	2.59 (1.01)	0.95 (2.21)	<.001
Office visit	4.50 (4.61)	4.86 (4.14)	.65

a Control period was defined as 1 year prior to visit 1.

b Post period was defined as 1 year after visit 5.

c *P* < .05 indicates significance.

The ROI was 0.49 for participants who completed all 5 visits (n = 70) and 1.34 for the higher-use participants (n = 22).

## Implications for Public Health

The READY study demonstrated that a CHW asthma home visiting intervention not only improves health outcomes and reduces indoor asthma triggers, but also reduces Medicaid costs for pediatric patients. Few studies evaluating the efficacy and cost-savings for payers of pediatric asthma home visiting have used participant medical claims and have instead relied on nonpayment measures (9). READY compared participants’ MassHealth claims from the control period and post period of the intervention and confirmed significant reductions in asthma-related emergency department visits and associated costs, both for all participants and for higher-use participants. Cost reductions were also observed for asthma hospitalization but were not significant. READY further demonstrated a positive ROI for higher-use participants, a finding which would be achieved for the entire participant sample with additional years of follow-up, as described in other studies (10). Actual health care savings may also be underestimated because many children with asthma have family members with asthma who were not tracked, but also may have benefitted from the intervention (11).

Our study has several limitations. First, the health outcome analysis was based on caregivers’ self-report, which may introduce bias. Second, the sample size of MassHealth claims analyzed was small because of continuous enrollment and the requirement for signed PSI forms. A planned analysis using a comparison group drawn from MassHealth claims could not be completed because MassHealth claims do not contain race/ethnicity fields. Lastly, pharmacy claims were incomplete and could not be included.

The READY study showed that CHW-led asthma home visiting programs can produce short-term Medicaid cost savings and more appropriate asthma care use. For example, the noted 63% reduction in emergency department visits by those with 2 or more emergency department visits in the control period demonstrated that these participants avoided, on average, more than 1 emergency department visit in the post intervention period. Because it was not possible to determine the specific intervention components driving these use reductions, this result underscores the importance of multitrigger multicomponent asthma home visits led by CHWs. Because many states have moved from fee-for-service payment structures toward value-based payments, the results of READY offer health care systems an opportunity for meeting the triple aim of improving health, improving quality, and reducing costs (12). READY advances the literature by demonstrating cost savings and positive ROI using participants’ Medicaid claims data for CHW-led asthma home visiting interventions integrated into medical home teams. The ability of asthma home visiting programs to achieve 2 of the components of the triple aim (improved health outcomes and improved quality of care) has been previously established (13); now READY has demonstrated that this intervention can also lead to better value.
